# Identification of Ellagic Acid from Plant *Rhodiola rosea* L. as an Anti-Ebola Virus Entry Inhibitor

**DOI:** 10.3390/v10040152

**Published:** 2018-03-27

**Authors:** Qinghua Cui, Ruikun Du, Manu Anantpadma, Adam Schafer, Lin Hou, Jingzhen Tian, Robert A. Davey, Han Cheng, Lijun Rong

**Affiliations:** 1 College of Pharmacy, Shandong University of Traditional Chinese Medicine, Jinan 250355, China; user753951@163.com (Q.C.); ruikun7128@gmail.com (R.D.); houlin5027@163.com (L.H.); tianjingzhen@163.com (J.T.); 2 Department of Microbiology and Immunology, College of Medicine, University of Illinois at Chicago, Chicago, IL 60612, USA; aschaf5@uic.edu; 3 Department of Virology and Immunology, Texas Biomedical Research Institute, San Antonio, TX 78227, USA; manantpadma@txbiomed.org (M.A.); rdavey@txbiomed.org (R.A.D.)

**Keywords:** Ebola, Marburg, *Rhodiola rosea*, ellagic acid, gallic acid, Traditional Chinese medicine

## Abstract

The recent 2014–2016 West African Ebola virus epidemic underscores the need for the development of novel anti-Ebola therapeutics, due to the high mortality rates of Ebola virus infections and the lack of FDA-approved vaccine or therapy that is available for the prevention and treatment. Traditional Chinese medicines (TCMs) represent a huge reservoir of bioactive chemicals and many TCMs have been shown to have antiviral activities. 373 extracts from 128 TCMs were evaluated using a high throughput assay to screen for inhibitors of Ebola virus cell entry. Extract of *Rhodiola rosea* displayed specific and potent inhibition against cell entry of both Ebola virus and Marburg virus. In addition, twenty commercial compounds that were isolated from *Rhodiola rosea* were evaluated using the pseudotyped Ebola virus entry assay, and it was found that ellagic acid and gallic acid, which are two structurally related compounds, are the most effective ones. The activity of the extract and the two pure compounds were validated using infectious Ebola virus. The time-of-addition experiments suggest that, mechanistically, the *Rhodiola rosea* extract and the effective compounds act at an early step in the infection cycle following initial cell attachment, but prior to viral/cell membrane fusion. Our findings provide evidence that *Rhodiola rosea* has potent anti-filovirus properties that may be developed as a novel anti-Ebola treatment.

## 1. Introduction

Ebola virus (EBOV) and Marburg virus (MARV) belong to the Filoviridae family and are enveloped, single-stranded, negative-sense RNA viruses with filamentous morphology. Infections by these viruses can cause severe hemorrhagic fevers in humans and nonhuman primates with mortality rates up to 90% [1]. The recent 2014–2016 Ebola epidemic led to more than 11,500 reported deaths, demonstrating the urgent medical need for effective anti-EBOV vaccines and therapeutics for humans [2].

Natural products are considered to be a natural combinatorial chemical source because they contain structurally diversified bioactive chemicals, thus making them a valuable reservoir for new drug discovery. Natural products or their direct derivatives account for 34% of new medicines approved by the US Food and Drug Administration (FDA) between 1981 and 2010 [3]. Traditional Chinese medicines (TCMs), which are mostly derived from natural products, have been used in China and other countries/regions to treat various diseases for more than 2000 years, and are now gaining popularity in Europe and North America as complementary alternative treatments. A growing number of TCMs have been proven to have antiviral activities with low toxicity and a few herbs have been approved by the China Food and Drug Administration to treat infections by viruses, including influenza virus, herpes simplex virus, hepatitis B virus, enterovirus, and respiratory syncytial virus [4]. Thus, TCMs represent an alternative source to identify novel antiviral therapeutics.

To identify new drug candidates from TCMs, we previously established a high throughput screen (HTS) platform to identify entry inhibitors for highly-pathogenic viruses [5]. This screening strategy targets the entry of viruses, which is determined by the glycoproteins (GP) on the virion surface. The native form of EBOV and MARV GP is a trimer of GP1/GP2 heterodimer, with GP1 responsible for receptor recognition and binding and GP2 for mediating viral/host membrane fusion in the endosome [6]. Interestingly, although the sequences of the EBOV and MARV GPs are quite divergent, they do share many conserved features in receptor recognition and membrane fusion, indicating the possibility to develop broad spectrum antivirals against both of the viruses [7,8]. For example, both EBOV and MARV GPs recognize the attachment factors, like glycosaminoglycans or C-type lectin, etc. and use macropinocytosis to internalize the virion, which is then trafficked through the endosomal-lysosomal system [9,10,11,12,13]. The GP is further cleaved by cysteine proteases cathepsin B and L to expose a region of GP that binds Niemann-Pick C1 (NPC1), the internal EBOV/MARV receptor [14,15,16,17,18]. The GP-NPC1 interaction is necessary for fusion of the virus membrane with the host endosomal membrane in the late endosome/lysosome to release the capsid to the cell cytoplasm and initiate genome replication [19,20]. Recently, a few compounds have been identified to target the EBOV-GP mediated virus entry, making it a promising target for drug development [18,21,22,23,24,25]. However, none of these promising leads has been developed into a treatment. Given that many leads do not make it past clinical advancement, additional treatment candidates are still needed.

In this report, we evaluated 373 TCM extracts and demonstrated that extracts of *Rhodiola rosea* and two isolated, chemically related compounds effectively inhibit Ebola virus entry. These findings have important implications in exploring and developing TCMs as potential antivirals against Ebola and Marburg viral infections.

## 2. Materials and Methods

### 2.1. Cell Culture

Human lung epithelial cell line A549, human embryonic kidney cell line 239T, and Hela cell line were cultured in DMEM (Cellgro, Manassas, VA, USA) supplemented with 10% fetal bovine serum (FBS, Gibco, Carlsbad, CA, USA), 100 μg/mL of streptomycin, and 100 units of penicillin (Invitrogen, Carlsbad, CA, USA) at 37 °C and 5% CO_2_.

### 2.2. Generation of Pseudovirions

HIV/MARV, HIV/EBOV, HIV/H5N1, and HIV/H7N1 pseudovirions were produced by transient co-transfection of a corresponding GP encoding plasmid (Influenza A—hemagglutinin (HA) and neuraminidase (NA) from A/Goose/Qinghai/59/05 (H5N1) strain or A/Netherlands/219/03 (H7N7) strain; Marburgvirus—GP; Ebolavirus Zaire—GP) and the HIV core plasmid (pNL4-3.Luc.R^−^E^−^) into 293T cells using a polyethylenimine based transfection protocol. Cells were washed with phosphate-buffered saline (PBS) 6 h after transfection, and 20 mL fresh media was added to each Falcon^®^ 150 mm TC-treated cell culture dish. The supernatants were collected and filtered through 0.45 μm pore size filter (Nalgene, Rochester, NY, USA) 24 h post-transfection and the pseudovirion stocks were stored at 4 °C prior to use. The H7N1 pseudoviruses were activated with 10 µg/mL TPCK-trypsin before infecting A549 cells

### 2.3. Preparation of TCM Extracts

One hundred and twenty-eight traditional Chinese herbs were purchased from the Chinese herbal medicine market in China. 373 extracts were prepared from the plants using extraction methods including aqueous extraction and organic-solvent extraction. They were dried in vacuum at 50 °C and dissolved in DMSO. Extracts were arrayed in a 384-well plate at a 20 mg/mL stock concentration in solvent. All of the sample plates were stored at −20 °C until use. Twenty chemical components (>98% purity) from *R. rosea* were purchase from National Institutes for Food and Drug Control (Jinan, China).

### 2.4. High-Throughput Screen

The TCM extract library was screened in 384-well plates with a final concentration of 12.5 µg/mL in 0.0625% DMSO (*v/v*). Low-passage A549 cells were seeded in 384-well plates at a density of 1000 cells/well 24 h before infection. In the presence of TCM extracts, A549 cells were challenged with HIV/EBOV, HIV/H5N1, or HIV/H7N1 pseudotyped virions in JANUS robotic workstation (PerkinElmer, Waltham, MA, USA). Infection was quantified after 48 h incubation by measuring the luciferase activity with the neolite Reporter Gene Assay System (PerkinElmer). Data was normalized to signals from the negative controls (virus alone with DMSO) and an average >90% inhibition for duplicates was applied for picking hits.

The selected active extracts were then reformatted into new 96-well plates and tested against, HIV/EBOV, HIV/MARV, HIV/H5N1, or HIV/H7N1 at 12.5 µg/mL in 0.0625% DMSO (*v/v*) to confirm the primary results. Cell cytotoxicity was examined 48 h post treatment using the CellTiter-Glo^®^ Luminescent Cell Viability Assay (Promega, Madison, WI, USA), treated as for the antiviral screen. 

The hit extract was two-fold serially that was diluted for dose-response titration, and the IC_50_ and CC_50_ values were determined by fitting dose-response curves with four-parameter logistic regression to the data in GraphPad Prism software (version 5.02, La Jolla, CA, USA).

### 2.5. Time-of-Addition Experiment 

HIV/EBOV virion was incubated with A549 cells at 4 °C for 1 h and then the virus was removed. The cells were washed with ice cold PBS twice before fresh media was added and virus entry was initiated by shifting the temperature to 37 °C. At different time points of virus entry, *R. rosea* extract (4 µg/mL), gallic acid (30 µM), ellagic acid (30 µM), benztropine (25 µM, Santa Cruz Biotech, Dallas, TX, USA), heparin (10 µg/mL, Sigma-Aldrich, St. Louis, MO, USA), zidovudine (2 µM, Sigma-Aldrich), CA-074 (100 µM, Tocris Bioscience, Bristol, UK), or drug vehicle DMSO was introduced to assess their impact on virus entry (triplicate wells for each treatment at each time point). Virus infection was quantified 48 h post infection as described above.

### 2.6. Infectious Virus Assays

Experiments using recombinant infectious Zaire Ebola virus were performed in biosafety level 4 (BSL-4) facilities at the Texas Biomedical Research Institute. The infectious EBOV and MARV were produced according to previously described methods [26]. The Zaire EBOV Mayinga strain in the assay was a kind gift of Heinz Feldmann (NIH, Rocky Mountain Laboratory, Hamilton, MT, USA), and it has an insertion of green fluorescent protein (GFP) between the nucleoprotein (NP) and VP35 [27]. The virus was grown in Vero cells and the virus in the clarified supernatant was pelleted through a 20% sucrose cushion by centrifugation at 141,118× *g* for 2 h at 4 °C. The virus pellet was suspended in PBS and stored in aliquots at −80 °C until use. For infection assays, 4000 HeLa cells per well were grown overnight in 384-well tissue culture plates in DMEM culture medium that was supplemented with 10% FBS; the cells were infected by EBOV-GFP virus (MOI of 0.05 to 0.15) in the presence of TCM extract or effective compounds at various concentrations. Bafilomycin at a final concentration of 10 nM was used as a positive control treatment. All of the treatments were done in duplicates. After 24 h incubation, post-infection cells were fixed by immersing the plates in formalin for 24 h at 4 °C. Fixed plates were inactivated using formalin and brought out of the BSL-4. Formalin was then decanted and plates were washed twice with PBS. Cell nuclei were stained using Hoechst at 1:50,000 dilution and plates were imaged. Nuclei (blue) and infected cells expressing GFP (green) were counted using CellProfiler software version 2.1.1 (Broad Institute, Cambridge, MA, USA). Total number of nuclei (blue) was used as a check of cell function, with >30% reduction, indicating a growth arrest or toxicity and were noted during analysis.

## 3. Results

### 3.1. Extracts of Rhodiola rosea Specifically Block Entry of Ebola and Marburg Viruses

**Figure 1:**
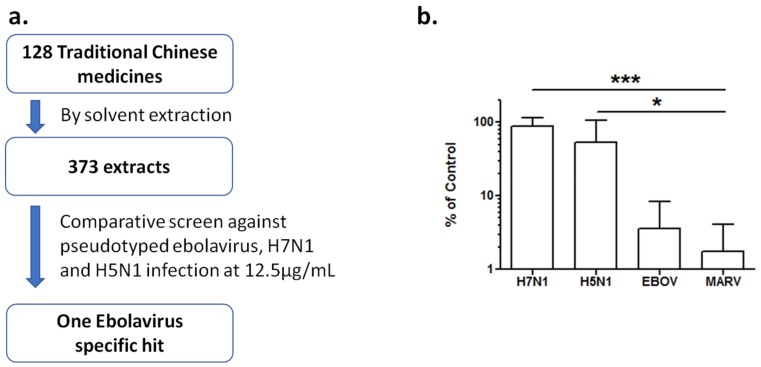
Identification of *R. rosea* extract as Ebola virus (EBOV) specific inhibitor. (**a**) A Traditional Chinese medicines (TCM) extract library of 373 extracts from 128 TCMs was screened against pseudoypted EBOV, H5N1, and H7N1 viruses at a final concentration of 12.5 μg/mL. Only *R. rosea* extract shows >90% inhibition against EBOV and <30% inhibitioin against H5N1 and H7N1 pseudovirions; (**b**) The *R. rosea* extract was further validated by various pseudovirions, including EBOV, MARV, H5N1, and H7N1 at a final concentration of 12.5 μg/mL. Data are means ± SD from three independent experiments. * *p* < 0.05, EBOV, MARV as compared to H5N1 group; *** *p* < 0.001, EBOV, MARV when compared to H7N1 group.

Natural products, including Traditional Chinese medicines (TCMs), represent a rich source of structurally diversified bioactive compounds in drug discovery. To find potential antivirals from TCMs, we first made a library of 373 crude extracts from 128 TCMs by aqueous extraction or organic-solvent extraction. With a previously established cell-based HTS platform [5], we performed a comparative screen of this library against three pseudotyped viruses to identify entry inhibitors. The TCM extract library was screened at a final concentration of 12.5 µg/mL against infection of HIV/EBOV, HIV/H7N1, and HIV/H5N1 pseudovirions in A549 cells. One EBOV specific hit was identified, which showed >90% inhibition against HIV/EBOV infection, but <30% inhibition against the infection of both and HIV/H7N1 and HIV/H5N1 pseudovirions (Figure 1a). This extract (from *Rhodiola rosea*) was further evaluated for cell toxicity and antiviral activity against HIV/EBOV, HIV/MARV, HIV/H5N1, and HIV/H7N1 pseudovirions at 12.5 µg/mL. The extract exhibited specific antiviral activity against both pseudotyped EBOV and MARV entry, but little inhibitory activity against the entry of the two pseudotyped influenza virus subtypes (Figure 1b), indicating that TCM plant *R. rosea* has broad activity against these distantly related filoviruses.

### 3.2. Anti-Filovirus Activities of the R. rosea Extracts by Different Extraction Methods

**Figure 2:**
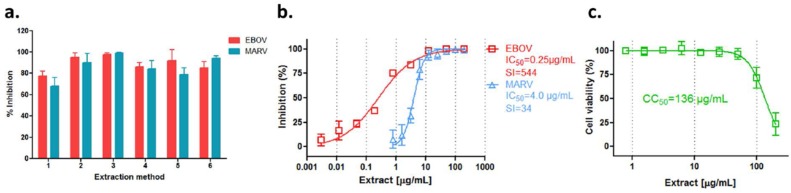
Anti-filovirus activities of *R. rosea* extracts. (**a**) Six extracts from *R. rosea* prepared by various extraction methods (Table 1) were evaluated against pseudotyped EBOV infection at 12.5 μg/mL; (**b**) The in vitro dose-response curves of *R. rosea* extract are shown against HIV/EBOV (□, red) and HIV/MARV (∆, blue) pseudovirion infections and (**c**) in cell toxicity assay (□, green) in A549 cells. Data are means ± SD (*n* = 3) from three independent experiments.

Since different extraction methods can greatly influence the composition and concentration of the effective bioactive molecules in the extracts, we compared the anti-filovirus activities of six different extracts of *R. rosea* by various extraction methods (Table 1). The six extracts were tested in A549 cells at 12.5 µg/mL against pseudotyped EBOV and MARV infection, with each extract showing strong antiviral activity against both viruses (Figure 2a). Extract 3 from ethanol ultrasonic extraction showed the best antiviral activity with 98% inhibition against HIV/EBOV and 99% inhibition against HIV/MARV, respectively. Extract 3 was further evaluated by titration against pseudotyped filoviruses. It demonstrated a 50% inhibitory concentration (IC_50_ value) of 0.25 µg/mL against EBOV pseudovirion, and an IC_50_ value of 4.0 µg/mL against the MARV pseudovirion, respectively (Figure 2b). The cytotoxicity of extract 3 was low, giving a 50% cytotoxic concentration (CC_50_ value) of 136 µg/mL (Figure 2c). The selectivity index (SI) of extract 3 was >500 for EBOV and 34 for MARV pseudovirion, thus making it an attractive candidate for further anti-filovirus evaluation and development.

### 3.3. Ellagic Acid and Gallic Acid of R. rosea Block Ebola-GP Mediated Viral Entry 

**Figure 3:**
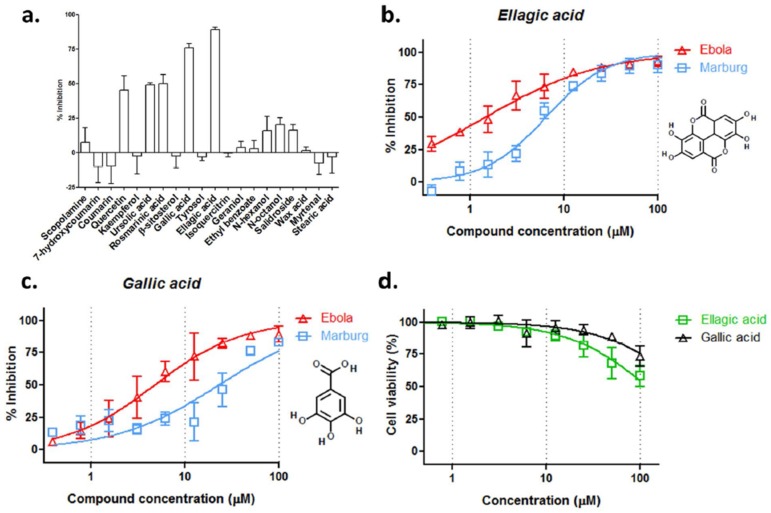
Identification of ellagic acid and gallic acid as the most active components of *R. rosea* against EBOV infection. (**a**) Twenty known *R. rosea* components were tested against pseudotyped EBOV infection at 12.5 μM; (**b**) Ellagic acid and (**c**) gallic acid were examined against HIV/EBOV (∆, red) and HIV/MARV (□, blue) infection in A549 cells in vitro for dose-response evaluation; (**d**) Cell viability assay of ellagic acid and gallic acid in A549 cells. Data are means ± SD (*n* = 3) from three independent experiments. The IC_50_/CC_50_ Values, 95% confidence intervals and selectivity indices for ellagic acid and gallic acid are listed in Supplementary Materials Table S1.

The chemical composition of *R. rosea* has been extensively studied and different chemical fractions are available, including saliroside, its aglycon tyrosol, cinnamic glycosides, flavonoids, tannins, gallic acid, as well as the essential oil. Twenty commercially available fractions were tested against HIV/EBOV infection in A549 cells at 12.5 µM. Ellagic acid and gallic acid were the most active against pseudotyped virion entry, which inhibited ~90% and ~80% EBOV entry, respectively. The less active components include quercetin, ursolic acid, and rosmarinic acid, which showed ~50% inhibition against the virus entry (Figure 3a).

Ellagic acid and gallic acid were further characterized. Each showed typical S-shaped dose-response inhibition against infection by both filoviral pseudovirions. Ellagic acid had an IC_50_ value of 1.4 µM and 6.4 µM against EBOV and MARV pseudovirions, respectively (Figure 3b). Gallic acid showed an approximately four-fold lesser activity against each pseudovirion (Figure 3c). Both compounds displayed negligible toxic effect in A549 cells; the CC_50_ for ellagic acid and gallic acid were 122 µM and 309 µM, respectively (Figure 3d). These results suggest that ellagic acid and gallic acid are likely major active components that contribute to the anti-filoviral activity of *R. rosea*.

### 3.4. R. rosea Extract, Ellagic Acid and Gallic Acid Inhibit Infectious EBOV Infection

**Figure 4:**
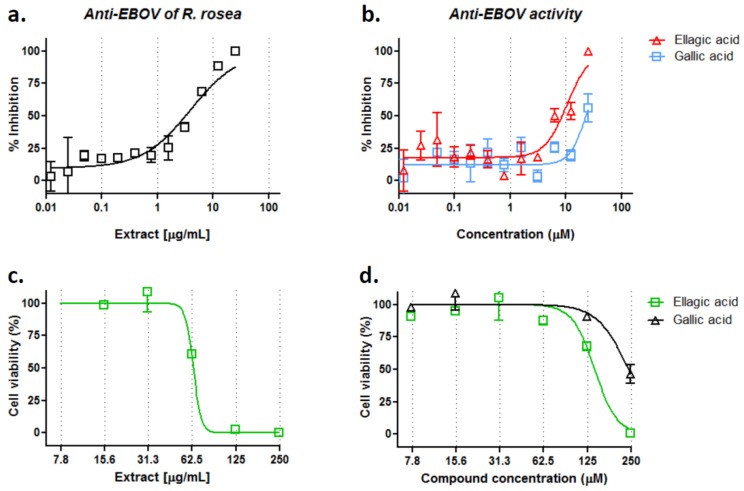
Anti-filovirus activities of *R. rosea* extract, ellagic acid and gallic acid against infectious EBOV. Dose-response titrations were evaluated in Hela cells against recombinant infectious EBOV-green fluorescent protein (GFP) infection for (**a**) *R. rosea* extract and (**b**) ellagic acid and gallic acid. Cell viability assays of (**c**) *R. rosea* extract and (**d**) ellagic acid and gallic acid were performed in Hela cells. Data are means ± SD (*n* = 2).

To validate the anti-filovirus activity of *R. rosea* extract and its active components, *R. rosea* extract (ethanol ultrasonic extraction), ellagic, and gallic acid were evaluated in HeLa cells against infectious EBOV infection in a BSL-4 facility. As shown in Figure 4a,b, *R. rosea* extract, ellagic acid, and gallic acid displayed good inhibition against infectious EBOV in HeLa cells. *R. rosea* extract showed an IC_50_ value of 3.9 µg/mL, ellagic acid, and gallic acid showed an IC_50_ value of 10.5 µM and 25.4 µM, respectively. The cell toxicity of *R. rosea* extract, ellagic acid, and gallic acid were also evaluated in HeLa cells, with CC_50_s at 64 µg/mL, 141 µM, 241 µM, and selectivity indices at 16.4, 13.4, and 9.5, respectively. The antiviral activities were also examined against infectious MARV in HeLa cells, but only *R. rosea* extract exhibited ~75% inhibition at 25 µg/mL, the highest concentration tested (Supplementary Materials Figure S1). These results demonstrate that the *R. rosea* extract, ellagic acid, and gallic acid can effectively block infection of EBOV, while they are less effective against MARV.

### 3.5. R. rosea Blocks EBOV Infection at a Late Stage of Virus Entry

**Figure 5:**
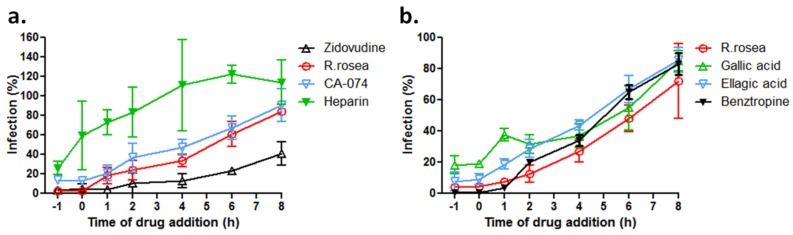
*R. rosea* extract, ellagic acid and gallic acid inhibit EBOV infection at late entry step. Pseudotyped HIV/EOBV was incubated with A549 cells at 4 °C at the −1 h time point. After 1 h of incubation, the virus was removed and temperature was shifted to 37 °C to trigger virus internalization. (**a**) *R. rosea* extract (4 µg/mL), heparin (10 µg/mL), zidovudine (2 µM), CA-074 (100 µM) were introduced at different time points of virus infection, and the compounds’ effects on viral infection are shown (means ± SD (*n* = 3)); (**b**) *R. rosea* extract (4 µg/mL), gallic acid (30 µM), ellagic acid (30 µM), and benztropine (25 µM) were introduced at different time points of virus infection, and the compounds’ effects on viral infection are shown (means ± SD (*n* = 3)).

To investigate at which entry stage that *R. rosea* extract and its active components exert antiviral activity, a “time-of-addition experiment”, as previously described [22], was performed with HIV/EBOV pseudovirions in the presence of *R. rosea* extract (4 µg/mL), ellagic acid (30 µM) or gallic acid (30 µM), and several control compounds. HIV/EBOV virions were allowed to attach to A549 cells at 4 °C and unattached virus was washed free. Cell uptake of virus was then initiated by shifting the incubation temperature to 37 °C. TCM extract or compounds were introduced at various time points to evaluate their antiviral effects. As shown in Figure 5a, heparin (10 µg/mL), exhibited the best antiviral effect at time point −1 h and quickly lost activity after virus entry started, indicating that it blocked virus attaching to the cell surface; zidovudine (2 µM) remained active until the late phase of virus entry, because it targets HIV reverse transcription, an event following viral/endosome fusion. *R. rosea* extract showed a similar trend as the cathepsin B inhibitor CA-074 (100 µM), but not that of heparin or zidovudine, suggesting that it may act at the same site as the cathepsin B inhibitor in the endosome. *R. rosea* extract and the two active components were also evaluated with benztropine. Benztropine started to lose its inhibitory effect when it was added at 1 h post entry and beyond, as consistent with its reported inhibition of EBOV-GP mediated fusion [28]. *R. rosea* extract, ellagic, and gallic acid each showed a similar time of loss of antiviral activity trend as benztropine, indicating that they may act at a similar post-attachment step, as benztropine, which blocks the infection of EBOV prior to viral/endosome membrane fusion (Figure 5b).

## 4. Discussion

In this study, we evaluated several hundred extracts of Traditional Chinese medicines (TCMs) in an effort to identify potential antiviral agents against filoviruses and other emerging viral pathogens, and showed that extracts of the plant *Rhodiola rosea* L. can specifically inhibit the entry and infection of Ebola and Marburg viruses. Further, we have identified that two chemically related compounds, gallic acid and ellagic acid isolated from *R. rosea* and commercially available, can effectively block entry of Ebola virus, and to a lesser extent, Marburg virus. Our findings demonstrate that *R. rosea*, and likely other TCMs, has strong anti-EBOV activities, and should be further explored and developed as potential antivirals.

Since the glycoprotein (GP) of Ebola (and Marburg) virus is the only viral surface protein that is responsible for binding to the surface receptor(s) and mediating the fusion of viral/cell membranes in the endosomal compartments [6], it has been considered a good anti-filoviral target. Indeed, GP has been a major target for developing prophylactic and therapeutic anti-Ebola vaccines, and several of potential vaccines have been shown to be highly effective in protecting animals (both small animals and non-human primates) against lethal Ebola virus infections, demonstrating that GP is an excellent antiviral target [29]. Similarly, several small molecule entry inhibitors of Ebola virus have been reported, including nonspecific cathepsin inhibitors and specific cathepsin B/L inhibitors [30,31,32,33,34,35,36], compounds targeting NPC1-Ebola GP interaction [18,24], and recently the GPCR antagonists (particularly, antihistamine-like inhibitors) [2,22]. However, whether any of these small molecule entry inhibitors is protective in animal models against lethal Ebola virus infection is not known yet. Therefore, it is imperative to explore and identify novel small molecules that are potent entry inhibitors as potential antivirals.

In contrast to synthetic small molecules, TCMs (and natural products) have better coverage of biologically relevant chemical space and are more likely to reach the intracellular site of action [3,37]. In fact, natural products, including TCMs, are one of the most important sources for discovery and development of new drugs. Many plant-derived compounds were reported with antiviral effects, and some of them have been investigated for their antiviral efficacy in animal studies and human clinical trials. Between 1981 and 2014, approximately 70% of approved antiviral small molecule drugs were originally derived from botanic natural products or their derivatives [38]. The current study focused on evaluating TCMs to identify potential anti-Ebola agents, using a cell-based screening high-throughput protocol developed by us [5,39], and we showed that *R. rosea* extract is an effective anti-filoviral agent, particularly against Ebola virus. *R. rosea* extract had an IC_50_ value of 0.25 µg/mL in the pseudovirus infection assay and an IC_50_ value of 3.9 µg/mL in an infectious EBOV infection assay, respectively, while it inhibited Marburg virus with an IC_50_ value of 4.0 µg/mL in pseudovirus assay and an IC_50_ value of ~25 µg/mL in infectious virus assay, respectively (Figure 2 and Figure 4). The anti-EBOV activity of *R. rosea* is better than that (IC_50_ ~6.25 µg/mL) of the best reported TCM *Prunella vulgaris* extract so far [40].

*R. rosea* has been extensively characterized and more than 140 chemicals have been identified, and roughly 50 polar compounds can be isolated from the water alcoholic extracts, including glycosides, organic acids, and phenolics [41]. Here, we show that two chemically related compounds, ellagic acid and gallic acid, are the most effective inhibitors among the 20 tested compounds, which were isolated from *R. rosea* (Figure 3). In addition, quercetin, ursolic acid, and rosmarinic acid also exhibited mild inhibition against pseudotyped EBOV entry, while the most studied components of *R. rosea,* such as salidroside and tyrosol, did not show anti-EBOV activity (Figure 3a). Ellagic acid showed roughly two-fold better anti-filovirus activity than gallic acid against the infectious recombinant EBOV infection, with IC_50_ values of 10.5 µM and 25.4 µM, respectively (Figure 4). When considering that the IC_50_ of ellagic and gallic acids are not much lower than that of the *R. rosea* extract, we conclude that other components (compounds) in the extract are involved in blocking Ebola virus entry in an additive or synergistic manner with ellagic acid and gallic acid. In addition, the much less anti-MARV activities of both the acids than the *R. rosea* extract also suggest that other component(s) may target glycoprotein in a virus-specific way. Further *R. rosea* fractionation studies are needed to identify and characterize those components.

Mechanistically, how the extracts of *R. rosea*, ellagic acid and gallic acid block Ebola virus entry is not clear at present. Gallic acid has been reported to inhibit the entry of human rhinoviruses and the Herpes Simplex Virus type 1 virus by preventing virion attachment to the cell surface [42,43]. However, this is unlikely to be the underlying inhibitory mechanism(s) on Ebola virus. Our studies (Figure 5a) showed that, unlike heparin, which blocks filovirus attaching to cell surface, introducing gallic acid or *R. rosea* extract after EBOV binding to the cell surface (after time point 0 h) still strongly inhibited EBOV entry, suggesting that the binding step is not the primary target by gallic acid or *R. rosea* extract [9]. The time-of-addition experiment suggests that these inhibitors act at a similar post-binding step in the endosome as cathepsin B inhibitor CA-074 or a previously characterized entry inhibitor benztropine that binds to the EBOV-GP protein and interferes with the GP-mediated fusion in the endosome [22,28]. Further, more delicate experiments are required to confirm this finding and pinpoint the exact entry step as the drug target. Another possibility is that the compounds from *R. rosea* target endosomal calcium channels, called two pore channels (TPCs), like tetrandine, which is a bio-benzylisoquinoline alkaloid isolated from a TCM plant, *Stephania tetrandra* [44]. These findings provide impetus for further exploring TCMs and natural products as potential antivirals against Ebola virus and other viruses. Because TCMs are more economically accessible and easier to store and distribute than a few therapies with preclinical data that include recombinant vesicular stomatitis virus-vectored/chimpanzee adenovirus type 3-vectored ebolavirus vaccine, and the expensive monoclonal antibody cocktail ZMapp, they represent a unique treatment option for the people in the resource-constrained Africa, where Ebola Virus outbreaks occur often. TCMs can be obtained cheaply and fast, so it could be used as complementary treatment to the vaccine/antibody based strategies when they are not immediately available.

In conclusion, we have identified *R. rosea* as a potent EBOV entry agent. This TCM has potent anti-Ebola activity and proven safety profile, making it a promising antiviral for further development. Though gallic and ellagic acids have been identified as active components, further detailed fractionation and chemical research is needed to purify other active ingredients and to elucidate how these chemicals act synergically in inhibiting filovirus entry.

## Figures and Tables

**Table 1:** Extraction methods for *Rhodiola Rose.*

No.	Extraction Method
1	water decoction followed by 70% ethanol precipitation
2	water extraction
3	70% ethanol ultrasonic extraction
4	water decoction
5	70% ethanol reflux extraction
6	water decoction followed by 60% ethanol precipitation

## Supplementary Materials

The following are available online at http://www.mdpi.com/1999-4915/10/4/152/s1, Figure S1: Anti-filovirus activities of *R. rosea* extract, ellagic acid and gallic acid against infectious MARV entry, Table S1: IC_50_/CC_50_ Values, 95% Confidence Intervals (CIs) and Selectivity indices (SIs) for Figure 3b–d.
