# Supplementary material for: Identification of Ellagic Acid from Plant Rhodiola rosea L. as an Anti-Ebola Virus Entry Inhibitor
Source: Viruses. 2018 Mar 27;10(4):152. doi: 10.3390/v10040152 (PMC5923446; doi:10.3390/v10040152)
Supplement: Supplementary file 1 [file viruses-10-00152-s001.pdf]

Supplementary Materials: Identification of Ellagic Acid from Plant *Rhodiola rosea* L. as an Anti-Ebola Virus Entry Inhibitor

Table S1. IC_{50}/CC_{50} Values, 95% Confidence Intervals (CIs) and Selectivity indices (SIs) for Figure 3b–d.

	EBOV IC_{50} (μM)	95% CI (μM)	MARV IC_{50} (μM)	95% CI (μM)	CC_{50} (μM)	95% CI (μM)	EBOV SI	MARV SI
Ellagic acid	1.44	1.19–1.75	6.38	5.44–7.47	121.6	101.8–145.3	84	19
Gallic acid	4.73	3.76–5.95	22.8	15.9–32.7	308.7	236.3–403.3	65	14

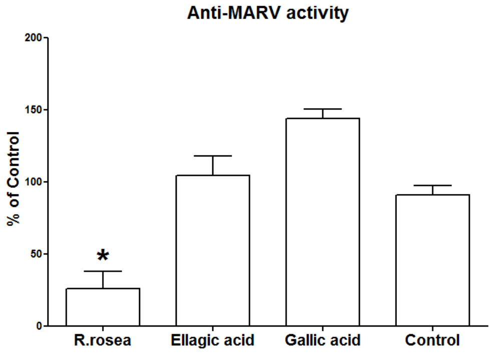

Figure S1. Anti-filovirus activities of *R. rosea* extract, ellagic acid and gallic acid against infectious MARV entry. *R. rosea* extract, ellagic acid and gallic acid were tested against infectious MARV infection at 25 μg/mL, 25 μM, and 25 μM respectively. The result was normalized by DMSO vehicle control and shown (means ± SD (n=2)). * $P<0.05$, *R. rosea* compared to control (untreated) group.
